# 4-Amino-3-ammonio­pyridinium dichloride

**DOI:** 10.1107/S1600536808041962

**Published:** 2008-12-17

**Authors:** Jian-Hua Qin, Jian-Ge Wang

**Affiliations:** a College of Chemistry and Chemical Engineering, Luoyang Normal University, Luoyang 471022, People’s Republic of China

## Abstract

The anions and cations of the title compound, C_5_H_9_N_3_^2+^·2Cl^−^, are connected by two chloride-bridged three-centered N—H⋯Cl hydrogen bonds into a three-dimensional network. The aromatic rings are not involved in stacking inter­actions.

## Related literature

For bond distances and angles in pyridine, derived from microwave spectra, see: Sørensen *et al.* (1974 ▶). For details of the N—H⋯Cl hydrogen bond in 4,4′-bipyridine compounds, see: Iyere *et al.* (2003 ▶). For N—H⋯Cl and secondary inter­actions in pyridinium chlorides, see: Jones *et al.* (2002 ▶); in 4-acetyl­pyridinium chloride, see: Kochel (2005 ▶). For N—H⋯Cl and O—H⋯Cl contacts in a triphenyl-pyridinium chloride (1/1) adduct, see: Sykora & Cioffi (2007 ▶).
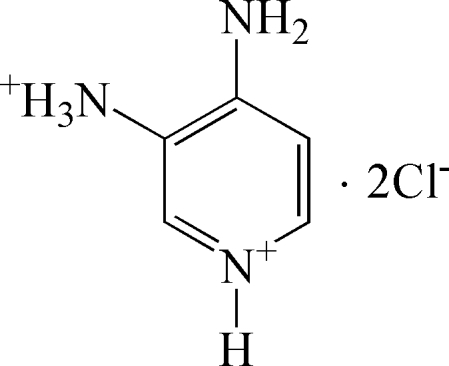


## Experimental

### 

#### Crystal data

C_5_H_9_N_3_^2+^·2Cl^−^
*M* *_r_* = 182.05
Monoclinic, 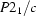

*a* = 8.362 (2) Å
*b* = 7.3218 (19) Å
*c* = 13.239 (3) Å
β = 92.065 (4)°
*V* = 810.0 (4) Å^3^
*Z* = 4
Mo *K*α radiation
μ = 0.73 mm^−1^
*T* = 296 (2) K
0.41 × 0.31 × 0.07 mm

#### Data collection

Bruker SMART CCD area-detector diffractometer
Absorption correction: multi-scan (*SADABS*; Bruker, 1997 ▶) *T*_min_ = 0.734, *T*_max_ = 0.948
3949 measured reflections
1494 independent reflections
1345 reflections with *I* > 2σ(*I*)
*R*_int_ = 0.014

#### Refinement

*R*[*F* ^2^ > 2σ(*F* ^2^)] = 0.025
*wR*(*F* ^2^) = 0.068
*S* = 1.14
1494 reflections
92 parameters
H-atom parameters constrained
Δρ_max_ = 0.24 e Å^−3^
Δρ_min_ = −0.25 e Å^−3^

### 

Data collection: *SMART* (Bruker, 1997 ▶); cell refinement: *SAINT* (Bruker, 1997 ▶); data reduction: *SAINT*; program(s) used to solve structure: *SHELXS97* (Sheldrick, 2008 ▶); program(s) used to refine structure: *SHELXL97* (Sheldrick, 2008 ▶); molecular graphics: *SHELXTL* (Sheldrick, 2008 ▶); software used to prepare material for publication: *SHELXTL*.

## Supplementary Material

Crystal structure: contains datablocks I, global. DOI: 10.1107/S1600536808041962/si2142sup1.cif

Structure factors: contains datablocks I. DOI: 10.1107/S1600536808041962/si2142Isup2.hkl

Additional supplementary materials:  crystallographic information; 3D view; checkCIF report

## Figures and Tables

**Table 1:** Hydrogen-bond geometry (Å, °)

*D*—H⋯*A*	*D*—H	H⋯*A*	*D*⋯*A*	*D*—H⋯*A*
N1—H1*A*⋯Cl2^i^	0.89	2.22	3.1142 (15)	178
N1—H1*B*⋯Cl2^ii^	0.89	2.37	3.1754 (16)	151
N1—H1*C*⋯Cl1^iii^	0.89	2.23	3.0790 (16)	160
N2—H2*A*⋯Cl1^ii^	0.86	2.39	3.2188 (17)	163
N2—H2*B*⋯Cl1^iv^	0.86	2.42	3.2672 (17)	168
N3—H3⋯Cl2	0.86	2.59	3.2499 (16)	135
N3—H3⋯Cl2^v^	0.86	2.70	3.3198 (16)	130

Symmetry codes: (i) 
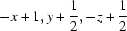
; (ii) 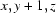; (iii) 
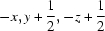
; (iv) 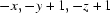; (v) 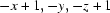.

**Fig. 1.**
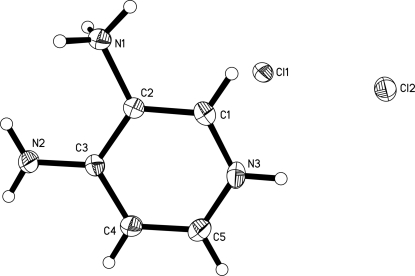
A view of the asymmetric unit of (I), showing the atom-labelling scheme. Displacement ellipsoids are drawn at the 30% probability level.

**Fig. 2.**
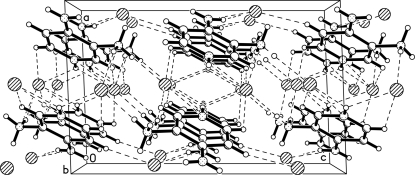
A view of the title compound packing down the b axis.

## supplementary crystallographic information

### Comment

The title compound is a salt containing a diprotonated 3,4-diaminopyridine cation and two Cl^-^ anions (Fig. 1). The C1—N3—C5 bond angle is wider than that in pyridine (116.94 (3)°; Sørensen *et al.*, 1974) which indicates that the pyridine ring N atom is protonated (Table 1). Also, the 4-amino N atom is protonated. The projection of the crystal packing along the *b* axis is shown in Fig. 2. The Cl^-^ anions and the 3,4-diaminopyridinium cations in the title compound are bonded by two chlorine-bridged, three-centered N—H···Cl hydrogen bonds into a three-dimensional network (Fig. 2, Table 2). Example structures of related compounds with two- and three-centered N—H···Cl hydrogen bonds are discussed by Iyere *et al.* (2003); Jones *et al.* (2002); Kochel (2005) and Sykora & Cioffi (2007).

### Experimental

3,4-diaminopyridine (0.01 mmol) and HCl (0.02 mmol) in 10 ml ethanol. Suitable crystals for X-ray analysis, were grown by allowing the solution to slowly evaporate for 15 days, and were subsequently filtered off, washed with methanol and dried under air.

### Refinement

H atoms were constrained to idealized positions and refined using a riding model, with C—H distances of 0.93 Å [*U*_iso_(H) = 1.2*U*_eq_(C)], and NH distances of 0.86 Å for NH2 [*U*_iso_(H) = 1.2*U*_eq_(N)] and 0.89 Å for NH3 [*U*_iso_(H) = 1.5*U*_eq_(N)].

### Crystal data

**Table:**

C_5_H_9_N_3_^2+^·2Cl^−^	*F*(000) = 376
*M**_r_* = 182.05	*D*_x_ = 1.493 Mg m^−^^3^
Monoclinic, *P*2_1_/*c*	Mo *K*α radiation, λ = 0.71073 Å
Hall symbol: -P 2ybc	Cell parameters from 2439 reflections
*a* = 8.362 (2) Å	θ = 3.1–28.2°
*b* = 7.3218 (19) Å	µ = 0.73 mm^−^^1^
*c* = 13.239 (3) Å	*T* = 296 K
β = 92.065 (4)°	Block, colorless
*V* = 810.0 (4) Å^3^	0.41 × 0.31 × 0.07 mm
*Z* = 4	

### Data collection

**Table:**

Bruker SMART CCD area-detector diffractometer	1494 independent reflections
Radiation source: fine-focus sealed tube	1345 reflections with *I* > 2σ(*I*)
graphite	*R*_int_ = 0.014
φ and ω scans	θ_max_ = 25.5°, θ_min_ = 2.4°
Absorption correction: multi-scan (*SADABS*; Bruker, 1997)	*h* = −10→9
*T*_min_ = 0.734, *T*_max_ = 0.948	*k* = −6→8
3949 measured reflections	*l* = −16→15

### Refinement

**Table:**

Refinement on *F*^2^	Primary atom site location: structure-invariant direct methods
Least-squares matrix: full	Secondary atom site location: difference Fourier map
*R*[*F*^2^ > 2σ(*F*^2^)] = 0.025	Hydrogen site location: inferred from neighbouring sites
*wR*(*F*^2^) = 0.068	H-atom parameters constrained
*S* = 1.14	*w* = 1/[σ^2^(*F*_o_^2^) + (0.0285*P*)^2^ + 0.2927*P*] where *P* = (*F*_o_^2^ + 2*F*_c_^2^)/3
1494 reflections	(Δ/σ)_max_ = 0.001
92 parameters	Δρ_max_ = 0.24 e Å^−^^3^
0 restraints	Δρ_min_ = −0.24 e Å^−^^3^

### Fractional atomic coordinates and isotropic or equivalent isotropic displacement parameters (Å^2^)

**Table:**

	*x*	*y*	*z*	*U*_iso_*/*U*_eq_	
Cl1	0.05806 (5)	0.10977 (6)	0.30663 (3)	0.03884 (15)	
Cl2	0.51526 (5)	0.00029 (6)	0.35359 (3)	0.03603 (14)	
N1	0.26647 (16)	0.68257 (19)	0.30328 (10)	0.0320 (3)	
H1A	0.3291	0.6281	0.2594	0.048*	
H1B	0.3026	0.7949	0.3160	0.048*	
H1C	0.1670	0.6887	0.2772	0.048*	
N2	0.11914 (19)	0.8105 (2)	0.48188 (12)	0.0439 (4)	
H2A	0.1182	0.8773	0.4284	0.053*	
H2B	0.0737	0.8485	0.5352	0.053*	
N3	0.33856 (18)	0.3102 (2)	0.48536 (11)	0.0376 (4)	
H3	0.3861	0.2061	0.4869	0.045*	
C1	0.3383 (2)	0.4103 (2)	0.40010 (13)	0.0329 (4)	
H1	0.3866	0.3649	0.3431	0.039*	
C2	0.26762 (18)	0.5778 (2)	0.39701 (12)	0.0271 (3)	
C3	0.19067 (19)	0.6488 (2)	0.48205 (12)	0.0296 (4)	
C4	0.1921 (2)	0.5357 (2)	0.56891 (13)	0.0366 (4)	
H4	0.1422	0.5751	0.6267	0.044*	
C5	0.2655 (2)	0.3703 (2)	0.56862 (14)	0.0391 (4)	
H5	0.2656	0.2976	0.6262	0.047*	

### Atomic displacement parameters (Å^2^)

**Table:**

	*U*^11^	*U*^22^	*U*^33^	*U*^12^	*U*^13^	*U*^23^
Cl1	0.0414 (3)	0.0428 (3)	0.0324 (2)	0.00951 (19)	0.00267 (18)	−0.00166 (18)
Cl2	0.0410 (3)	0.0298 (2)	0.0379 (2)	0.00388 (17)	0.01080 (18)	0.00231 (17)
N1	0.0335 (7)	0.0344 (8)	0.0283 (7)	0.0006 (6)	0.0044 (6)	−0.0002 (6)
N2	0.0617 (10)	0.0360 (8)	0.0348 (8)	0.0202 (8)	0.0147 (7)	0.0032 (7)
N3	0.0403 (8)	0.0265 (7)	0.0462 (9)	0.0089 (6)	0.0023 (7)	0.0007 (6)
C1	0.0319 (9)	0.0323 (9)	0.0346 (9)	0.0018 (7)	0.0031 (7)	−0.0054 (7)
C2	0.0260 (8)	0.0283 (8)	0.0270 (8)	−0.0013 (6)	0.0012 (6)	−0.0008 (6)
C3	0.0309 (8)	0.0274 (8)	0.0306 (8)	0.0029 (7)	0.0017 (7)	−0.0016 (7)
C4	0.0434 (10)	0.0377 (10)	0.0291 (9)	0.0069 (8)	0.0069 (7)	0.0021 (7)
C5	0.0459 (10)	0.0366 (10)	0.0349 (10)	0.0038 (8)	0.0013 (8)	0.0077 (8)

### Geometric parameters (Å, °)

**Table:**

N1—C2	1.458 (2)	N3—H3	0.8600
N1—H1A	0.8900	C1—C2	1.361 (2)
N1—H1B	0.8900	C1—H1	0.9300
N1—H1C	0.8900	C2—C3	1.416 (2)
N2—C3	1.326 (2)	C3—C4	1.417 (2)
N2—H2A	0.8600	C4—C5	1.358 (3)
N2—H2B	0.8600	C4—H4	0.9300
N3—C1	1.346 (2)	C5—H5	0.9300
N3—C5	1.353 (2)		
			
C2—N1—H1A	109.5	C2—C1—H1	119.9
C2—N1—H1B	109.5	C1—C2—C3	121.06 (15)
H1A—N1—H1B	109.5	C1—C2—N1	119.26 (14)
C2—N1—H1C	109.5	C3—C2—N1	119.65 (14)
H1A—N1—H1C	109.5	N2—C3—C2	122.95 (15)
H1B—N1—H1C	109.5	N2—C3—C4	120.92 (15)
C3—N2—H2A	120.0	C2—C3—C4	116.12 (15)
C3—N2—H2B	120.0	C5—C4—C3	120.64 (16)
H2A—N2—H2B	120.0	C5—C4—H4	119.7
C1—N3—C5	121.29 (15)	C3—C4—H4	119.7
C1—N3—H3	119.4	N3—C5—C4	120.64 (16)
C5—N3—H3	119.4	N3—C5—H5	119.7
N3—C1—C2	120.23 (16)	C4—C5—H5	119.7
N3—C1—H1	119.9		
			
C5—N3—C1—C2	1.9 (3)	N1—C2—C3—C4	177.72 (15)
N3—C1—C2—C3	−1.2 (2)	N2—C3—C4—C5	179.99 (18)
N3—C1—C2—N1	−178.99 (14)	C2—C3—C4—C5	0.7 (3)
C1—C2—C3—N2	−179.35 (16)	C1—N3—C5—C4	−1.2 (3)
N1—C2—C3—N2	−1.6 (2)	C3—C4—C5—N3	−0.1 (3)
C1—C2—C3—C4	−0.1 (2)		

### Hydrogen-bond geometry (Å, °)

**Table:**

*D*—H···*A*	*D*—H	H···*A*	*D*···*A*	*D*—H···*A*
N1—H1A···Cl2^i^	0.89	2.22	3.1142 (15)	178
N1—H1B···Cl2^ii^	0.89	2.37	3.1754 (16)	151
N1—H1C···Cl1^iii^	0.89	2.23	3.0790 (16)	160
N2—H2A···Cl1^ii^	0.86	2.39	3.2188 (17)	163
N2—H2B···Cl1^iv^	0.86	2.42	3.2672 (17)	168
N3—H3···Cl2	0.86	2.59	3.2499 (16)	135
N3—H3···Cl2^v^	0.86	2.70	3.3198 (16)	130

Symmetry codes: (i) −*x*+1, *y*+1/2, −*z*+1/2; (ii) *x*, *y*+1, *z*; (iii) −*x*, *y*+1/2, −*z*+1/2; (iv) −*x*, −*y*+1, −*z*+1; (v) −*x*+1, −*y*, −*z*+1.
